# Innovative Perspectives on Biofilm Interactions in Poultry Drinking Water Systems and Veterinary Antibiotics Used Worldwide

**DOI:** 10.3390/antibiotics11010077

**Published:** 2022-01-09

**Authors:** Friederike Hahne, Simon Jensch, Gerd Hamscher, Jessica Meißner, Manfred Kietzmann, Nicole Kemper, Jochen Schulz, Rafael H. Mateus-Vargas

**Affiliations:** 1 Institute of Food Chemistry and Food Biotechnology, Justus Liebig University Giessen, Heinrich-Buff-Ring 17, D-35392 Giessen, Germany; Friederike.Hahne@lcb.chemie.uni-giessen.de (F.H.); simon@jensch.eu (S.J.); Gerd.Hamscher@lcb.chemie.uni-giessen.de (G.H.); 2 Department of Pharmacology, Toxicology and Pharmacy, University of Veterinary Medicine Hannover, Foundation, Bünteweg 17, D-30559 Hannover, Germany; jessica.meissner@tiho-hannover.de (J.M.); manfred.kietzmann.iR@tiho-hannover.de (M.K.); rafaelh.mateusvargas@gmail.com (R.H.M.-V.); 3 Institute for Animal Hygiene, Animal Welfare and Farm Animal Behavior, University of Veterinary Medicine Hannover, Foundation, Bischofsholer Damm 15, D-30173 Hannover, Germany; jochen.schulz@tiho-hannover.de

**Keywords:** poultry production, flock treatment, sessile bacteria, sulfadiazine, trimethoprim, tylosin A

## Abstract

Prudent use of antibiotics in livestock is widely considered to be important to prevent antibiotic resistance. This study aimed to evaluate the interactions between biofilms and veterinary antibiotics in therapeutic concentrations administrated via drinking water through a standardized experimental setup. In this context, two biofilms formed by pseudomonads (*Pseudomonas* (*P.*) *aeruginosa* or *P*. *fluorescens*) and a susceptible *Escherichia* (*E.*) *coli* strain were developed in a nutrient-poor medium on the inner surface of polyvinyl chloride pipe pieces. Subsequently, developing biofilms were exposed to sulfadiazine/trimethoprim (SDZ/TMP) or tylosin A (TYL A) in dosages recommended for application in drinking water for 5 or 7 days, respectively. Various interactions were detected between biofilms and antibiotics. Microbiological examinations revealed that only TYL A reduced the number of bacteria on the surface of the pipes. Additionally, susceptible *E. coli* survived both antibiotic treatments without observable changes in the minimum inhibitory concentration to 13 relevant antibiotics. Furthermore, as demonstrated by HPLC-UV, the dynamics of SDZ/TMP and TYL A in liquid media differed between the biofilms of both pseudomonads over the exposure period. We conclude that this approach represents an innovative step toward the effective evaluation of safe veterinary antibiotic use.

## 1. Introduction

In recent decades, the increasing prevalence of antibiotic resistance observed among pathogens as well as commensal bacterial populations have thrust livestock systems into the limelight. In this matter, the use, but mostly the abuse, overuse, and misuse, of antibiotics in animal husbandry poses an increasing risk to public health [1]. The antibiotic-resistant microorganisms present within animal production may be transmitted to humans (e.g., food-borne, direct contact), other animals, and/or reach the environment [2]. The control of antimicrobial resistance development is a major challenge facing modern times as the number of antibiotic substances, which are still used to effectively treat severe bacterial infections, has decreased steadily in both human and veterinary medicine [1]. According to the World Health Organization [2], a key, unquestionable measure to appropriately mitigate the development and spread of antimicrobial resistance is the efficient usage of antimicrobial compounds at all levels in animal production, since antibiotic treatment is essential to treat diseases in animals and guarantee animal health.

In modern animal productions, the administration of drugs via drinking water is an important way to treat animals, particularly when large stocks require treatment [3]. Especially in poultry production, drinking water medication at the flock level is the preferred method due to the large numbers of animals, cost-efficacy, ease of administration, as well as immediate therapeutic care for all diseased or at-risk birds in the flock [4]. For administration in drinking water, a proven amount of the antimicrobial substance is injected into the water in proportion to the flock’s water consumption and distributed through a pipe network. To deliver the optimal dosage to each animal, the pharmacological properties of the antimicrobial compound must remain uncompromised in the drinking water, at least until it reaches the animals, and that sufficient concentrations are available for ingestion [5]. Before reaching the animals, antimicrobial compounds come into contact with biotic and abiotic factors present in the water distribution system. Due to their high biochemical activity and particular chemical-structural properties [6], biofilms represent an important entity among those factors. Biofilms are a network of microorganisms of different species surrounded by an external matrix of extracellular polymeric substances (EPS), which either adhere to a surface or move freely in a liquid medium. The EPS structure is very complex and includes polysaccharides, proteins, extracellular DNA, and metabolic products [7]. Once in a biofilm, sessile microorganisms possess different strategies, which allow them to tolerate and overcome conditions, which are noxious to planktonic cells, such as antimicrobial treatments. One important strategy of microorganisms to survive antibiotic exposure is to neutralize the activity of antimicrobial compounds [8]. Indeed, biofilms’ ability to transform, mineralize, or eliminate antimicrobial substances is currently part of the discussion around removing pharmaceutic micropollutants from wastewater (e.g., [9,10]). An additional characteristic of biofilm’s antimicrobial tolerance is the heterogeneous structure of the biofilm matrix, which provokes the establishment of spatial concentration gradients within biofilm layers [11]. The impaired diffusion of compounds permits at least some sessile cells to be exposed to sub-inhibitory concentrations and, thus, to survive [8]. Both concentration gradients [12] and the enhanced mutational status of bacterial cells in biofilms [13,14] make biofilms prominent sites for the development of antibiotic resistance [15].

From when drinking systems are put into operation, microorganisms colonize the inner surfaces of water lines and develop further into biofilms [16]. Once established in the pipe network, sessile bacteria are only affected to a very limited extent by cleaning and disinfection procedures [17,18], and consequently, close contact between antimicrobial compounds and biofilms as they traverse the pipe network before reaching the animals is unavoidable. Hence, the question arises if the interplay between biofilms proliferating on the inner surface of water distribution systems and the antibiotics administered via drinking water may influence the therapeutic outcome and/or contribute to the development of antibiotic resistance in animal husbandry. Recently, some groups have studied the effects of trace concentrations of emerging contaminants in residential drinking water distribution systems, including antibiotics, on the dynamics of biofilm formation as well as on susceptibility to antibiotics [19,20]. To the best of our knowledge, no studies have focused on the implications of the contact between antibiotics and biofilms under the conditions present in water lines serving animal premises, which includes exposure to therapeutic concentrations of antibiotics for up to several days. Since bacterial cells’ responses to antimicrobial pressure are in close conjunction with their environmental conditions [21], data currently available concerning biofilm–antibiotic interactions under different conditions do not allow direct transferability to drinking systems in animal husbandry.

Thus, this study aimed to experimentally evaluate for the first time whether the administration of antibiotics in therapeutic concentrations via drinking water may contribute to the development of antibiotic resistance in biofilm bacterial cells in drinking systems, and/or if the therapeutic efficacy is affected by biofilms. For this purpose, a model biofilm containing pseudomonads and *E. coli* was grown on polyvinyl chloride (PVC) surfaces under oligotrophic conditions. These biofilms were exposed to a combination of sulfadiazine/trimethoprim (SDZ/TMP) or tylosin A (TYL A). The investigations included daily monitoring of antimicrobial compounds’ behavior during contact with biofilms as well as the control of biofilm growth after exposure. Furthermore, the antimicrobial susceptibility of surviving *E. coli* from the biofilm was screened for their resistance status after antibiotic exposure.

## 2. Results

### 2.1. Bacterial Composition of Biofilms

**Figure 1:**
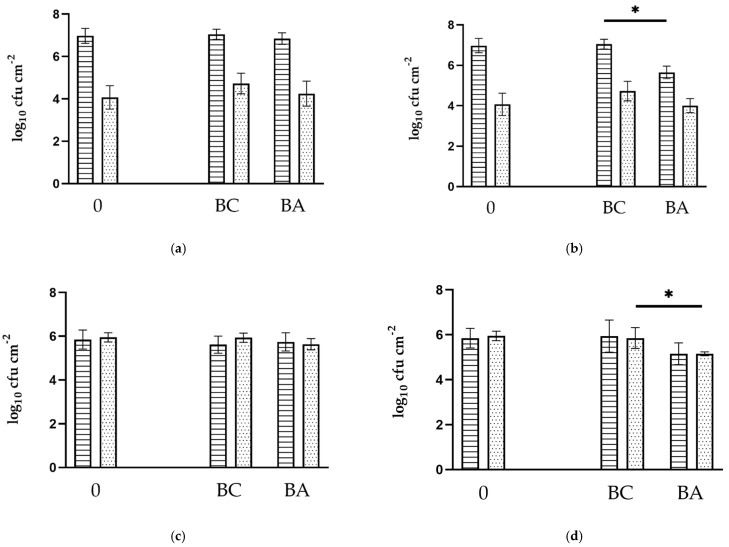
Population distribution (log_10_ cfu cm^−2^; means ± SD) of 7-day-old biofilms before (0) and after antibiotic application (BA) compared with controls stored the same period (BC). (**a**,**b**) Data for biofilms with *P. aeruginosa;* (**c**,**d**) Data for biofilms with *P. fluorescens.* (**a**,**c**) Results of exposure to SDZ/TMP (initial application: 150/30 mg L^−1^); (**b**,**d**) Results of exposure to TYL A (initial application: 500 mg L^−1^). Bars with striped and dotted patterns indicate log_10_-values for pseudomonads and *E. coli*, respectively. * Counts significantly different between exposed and control biofilms according to the *t*-test (**b**) *P. aeruginosa p* = 0.004; (**d**) *E. coli p* = 0.02, and Mann–Whitney test (**d**) *E. coli p* = 0.03.

Bacterial community distributions of dual-species biofilms observed in the different experiments are summarized in Figure 1. Microbiological examinations prior to antibiotic exposure showed that inoculated bacterial cells of pseudomonads and *E. coli* adhered and successfully formed a co-culture biofilm on the inner surface of PVC pipes under the conditions mentioned in the Materials and Methods (0; Figure 1). As observed for the controls, 7-day-old biofilms were on average composed by a predominant community of *P. aeruginosa* (6.98 ± 0.35 log_10_ cfu cm^−2^) and clearly less *E. coli* (4.07 ± 0.56 log_10_ cfu cm^−2^). In contrast, *P. fluorescens* and *E. coli* were determined in almost equal numbers on PVC surfaces after 7 days of growth (5.68 ± 0.44 log_10_ cfu cm^−2^ and 5.94 ± 0.24 log_10_ cfu cm^−2^, respectively). Regarding antibiotic exposure, bacterial population distribution in biofilms of PVC pipes was influenced differently depending on the antibiotic used. Although both pseudomonads and *E. coli* counts were slightly lower in exposed biofilms compared with controls, with differences between means of <0.5 log_10_ cfu cm^−2^, the exposure of 7-day-old biofilms to SDZ/TMP did not show any considerable variations regarding the bacterial composition (*p* > 0.05). In contrast, the application of TYL A resulted in an average difference between exposed and control biofilms of 1.40 and 0.72 log_10_ cfu cm^−2^ in the number of sessile *P. aeruginosa* and *E. coli* counts, and 0.79 and 0.70 log_10_ cfu cm^−2^ for *P. fluorescens* and *E. coli*, respectively (Figure 1). According to the *t*-test, the differences between biofilms exposed to TYL A and control biofilms were statistically significant for *P. aeruginosa* (*p* = 0.004) but not for *P. fluorescens* (*p* = 0.12). Regarding *E. coli,* exposure resulted in a statistical difference in biofilms with *P. fluorescens* (*p* = 0.02) but not in biofilms with *P. aeruginosa* (*p* = 0.11). Additionally, the comparison of ranks using the Mann–Whitney test only showed a statistical significance for the observed divergences between the cfu values of exposed *E. coli* and those of controls (*p* = 0.03) in biofilms with *P. fluorescens*.

### 2.2. Antimicrobial Susceptibility

A total of 20 *E. coli* isolates were obtained from biofilms exposed to SDZ/TMP and TYL A. The antibiotic exposure of co-culture biofilms did not cause any measurable changes in the minimum inhibitory concentration (MIC)-values of sessile *E. coli* isolates compared with controls. For all substances tested, the analyses of the MIC_50_ and MIC_90_ showed that the susceptibility did not differ between *E. coli* isolates prior to and after antibiotic exposure. The MIC_50_ and MIC_90_ of the isolates obtained from all of the exposed biofilms are summarized in Table 1.

### 2.3. Validation Parameters of the Analytical HPLC Method for SDZ, TMP, and TYL A

Limit of detection (LOD) and limit of quantification (LOQ) were determined using the calibration method as recommended by DIN 32645 [23]. For the standard solutions of SDZ and TMP, the LODs were 0.031 and 0.004 mg L^−1^ and the LOQs were 0.107 and 0.014 mg L^−1^. Linearity of SDZ was confirmed from LOQ to 200 mg L^−1^. For TMP, linearity was given for 10 to 100 mg L^−1^. The intraday precision of SDZ was 0.22% at 150 mg L^−1^ and TMP was 0.22% at 30 mg L^−1^. The LOD and LOQ of TYL A were 0.2 and 0.6 mg L^−1^. Linearity was confirmed from 25 to 150 mg L^−1^ and the intraday precision was 0.1% at 100 mg L^−1^.

### 2.4. Dynamics of Antibiotics

**Figure 2:**
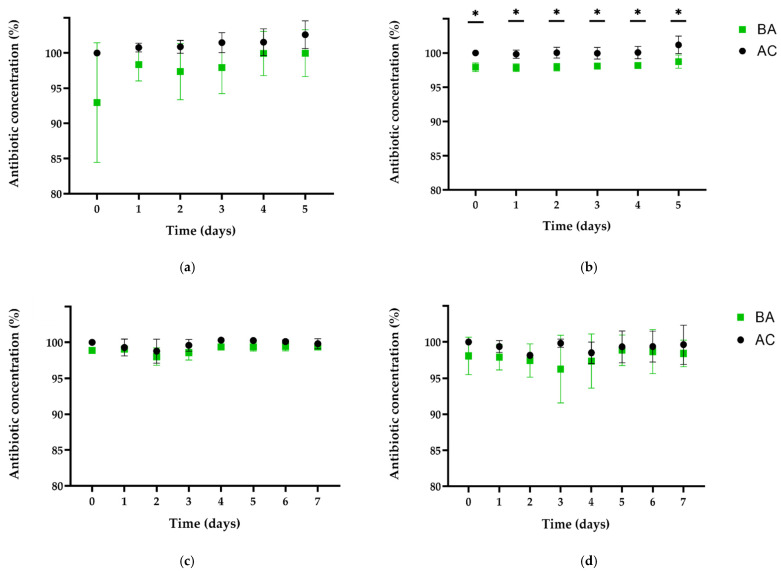
Antibiotic concentration (%) in the supernatant of pipes without biofilm (AC) or with 7-day-old biofilms (BA). Results are shown for daily samplings starting 1 h after application (0) over 5 or 7 days. (**a**,**b**) Data for biofilms with *P. aeruginosa;* (**c**,**d**) Data for biofilms with *P. fluorescens.* (**a**,**c**) Results for SDZ (initial application: 150 mg L^−1^); (**b**,**d**) Results for TMP (initial application: 30 mg L^−1^). * Concentrations significantly different between the antibiotics exposed to biofilms and control pipes (*p* ≤ 0.005).

Figure 2 shows the concentration of SDZ and TMP found in the supernatant of pipes containing biofilm (BA) compared with pipes without biofilm (AC) over the exposure period. Regarding our experiments with biofilms of *P. aeruginosa* and *E. coli*, it was determined already after 1 h (day 0) that the antibiotic content in supernatants was altered by confrontation with 7-day-old biofilms (Figure 2). In the following days, average concentrations of supernatants of BA remained in a range from 1.6 to 7.0% for SDZ and 1.9 to 2.4% for TMP lower than AC over 5 days. However, no degradation or transformation products related to these antibiotics were detected by HPLC-UV in the supernatant at any sampling time over the confrontation period. Due to the obvious uncertainty of measurement observed between the trials for SDZ, only the differences of TMP detected in supernatant between BA and AC were statistically significant using both statistical tests at sampling times T0 to T4 (*p* < 0.01). On day 5, there was a statistically significant difference in TMP concentration only with t-test (*p* = 0.005) (Figure 2). Moreover, examination of the PBS-surfactant solution samples by HPLC-UV revealed higher (7 to 30 times) residual concentrations of SDZ on BA surfaces compared with their AC counterparts. Similarly, lower concentrations of SDZ/TMP were detected after 1 h in the supernatants of pipes containing *P. fluorescens* and *E. coli* biofilms compared with controls. However, considering differences ranging from 0.8 to 1.3% for SDZ and 0.2 to 4.9% for TMP, measurements were more consistent between trials compared with biofilms containing *P. aeruginosa*. In addition, residual SDZ in PBS-solution was detected in BA (below LOQ) but not in AC (below LOD). Moreover, no transformation or degradation products were detectable for SDZ and TMP in the pipes containing biofilms.

**Figure 3:**
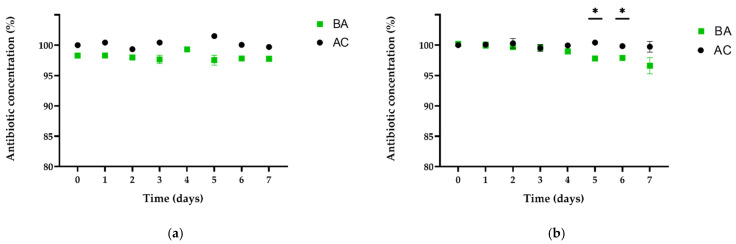
Antibiotic concentration (%) for TYL A in supernatants of pipes without biofilm (AC) or with 7-day-old biofilms (BA). Results are shown for daily samplings starting 1 h after application (0) over 7 days. (**a**) Data for biofilms with *P. aeruginosa;* (**b**) Data for biofilms with *P. fluorescens.* * Concentrations significantly different between antibiotics confronted to biofilms and control pipes (*t*-test; *p* < 0.05). T4 of the left figure (**a**) shows only data for BA.

**Figure 4:**
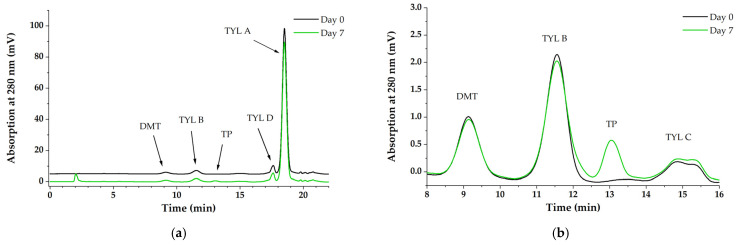
Comparative representation of HPLC-UV-chromatogram of BA with *P. fluorescens* exposed to TYL A at day 0 and day 7. (**a**) The overall outcome of UV-chromatogram; (**b**) Enlarged section of the UV-chromatogram showing concentration changes of TYL B and appearance of transformation product (TP) eluting between TYL B and TYL C.

**Figure 5:**
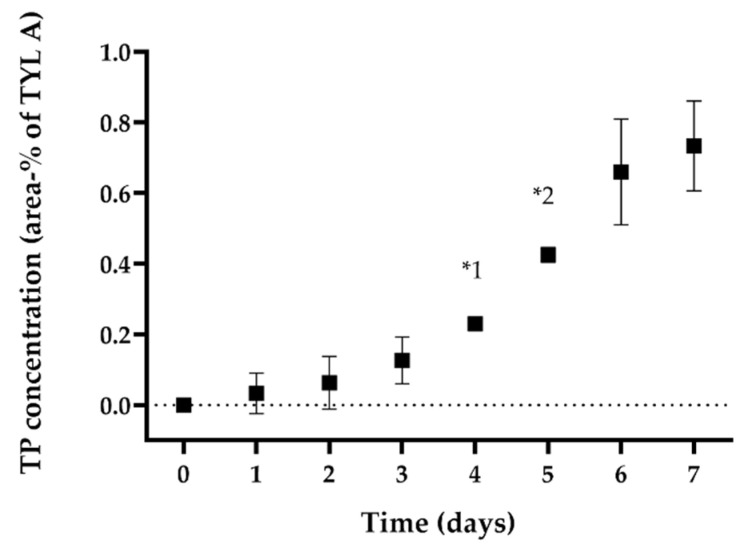
Amount of TP (area-% to TYL A) in the supernatant of pipes with 7-day-old biofilms with *P. fluorescens* (BA), results are shown with daily samplings starting 1 h after application (0) over 7 days. *1: one set with double sample collection (*n* = 1, N = 2), *2: two sets with double sample collection (*n* = 2, N = 4).

Regarding confronting *P. aeruginosa* biofilms with TYL A, the differences in concentration were similar to those observed for SDZ/TMP. In Figure 3, the concentration of TYL A is shown for supernatants of BA and AC over the exposure period. On average, TYL A concentration in BA was 1.7% lower compared with AC. Throughout the exposure period, the concentration of TYL A remained stable in the supernatant of both pipes. However, unlike the observations with SDZ/TMP, we were unable to detect any residual concentrations of TYL A on PVC surfaces with BA or those of AC. Contrary to these observations, the concentration of TYL A in the *P. fluorescens* BA supernatants was comparable with the concentration measured in the control pipe (AC) after 1 h of exposure (day 0). Interestingly, the concentration of TYL A in the pipes with BA decreased constantly in the following days and was at the end of the exposure period on average 3.4% lower than AC (Figure 3). During this time, it was possible to detect a new peak in the UV-chromatogram of supernatant of BA (between tylosin B and C; peak at ~13 min), which was not observed in the UV-chromatogram of pipes without biofilm (AC) at any sampling time. A comparison of the peaks observed in the UV-chromatogram at day 0 and day 7 of the exposure experiments in the supernatant of BA is shown in Figure 4. This observation indicated the increased accumulation of a transformation product in the supernatants of BA corresponding to the decrease in TYL A. The relative concentration of the transformation product (TP) at day 7 was 0.7 ± 0.1% compared with the total concentration of TYL A in BA at day 0. The development of the concentration of TP in the supernatant of BA in relation to the concentration of TYL A over the exposure period is shown in Figure 5. In this case, and similar to BA with *P. aeruginosa*, no residual TYL A or any of the TPs were detected in the PBS-Tween samples obtained from surfaces of pipes with biofilm (BA) or the controls (AC). An important aspect of TP is that, despite being stable in the sterile, filtered sample solution, after being refrigerated for several weeks (data not shown), further enrichment of TP was not possible. The latter was due to the low stability of TP after isolation in eluent solutions, which resulted in rapid reconfiguration toward the original TYL A. Thus, it was not possible to identify the TP’s molecular configuration. Another general difference observed between the BAs of both pseudomonads and AC was a peak elution at 2 min during the experiments in t BA samples. However, since this peak was also observed in all UV-chromatograms of biofilm samples without antibiotics (BC), this substance probably belongs to the polar components produced by bacteria in biofilms, i.e., pyoverdine by *P. fluorescens* [24].

## 3. Discussion

The administration of veterinary drugs via drinking water is an important way to treat livestock. Based on current literature, we hypothesized that biofilm–antibiotic interactions may have implications for the efficient usage of antimicrobial compounds and/or the development of antibiotic resistance. Although on-site studies would allow a real-life perspective of the interaction processes as well as the consequences, these first evaluations of biofilm–antibiotic interplay performed by our group, directly at an experimental model assembly of a drinking system (a description of which can be found in Mateus-Vargas et al. [16]), showed that this approach may still not be appropriate (data not shown). The latter is probably due to numerous influencing factors, which complicate accurate statements on the implications of such interactions. It is noteworthy that factors such as surface properties [25,26], availability of nutrients [27,28,29,30] as well as temperature [29,31,32] are reported to influence not only biofilm formation, but also biofilm response to disinfectants [27,33,34,35], and antibiotics [30,36]. Therefore, we developed an experimental in vitro method under conditions relevant to drinking water systems in animal premises. The selected conditions included oligotrophic media, temperatures ranging between 22 °C ± 1 °C, and the employment of pipes made of polyvinyl chloride (PVC), a material typically used in drinking systems of poultry houses. An important characteristic of the present approach is that a continuous flow of media was excluded from these experiments. As shown by others, hydraulics also influence the biofilm susceptibility of sessile bacteria to disinfectants [33]. Additionally, a continuous fluid shear accompanied by fresh nutrients supplies influenced the experimental outcome concerning the efficacy of several antibiotics on biofilms [37]. Although hydrodynamic conditions significantly influence biofilm development in drinking systems used in the poultry industry [16], flow characteristics in water lines of animal premises are not constant and vary depending mostly on the pipe network architecture as well as water consumption in the barn [3]. The latter also varies depending on several factors, including the number of animals, animal’s weight, as well as flock health status [5]. Considering all of these crucial factors, in conjunction with the application of therapeutic concentrations and recommended time period of administration, the present static experimental system provides basic assessments regarding the nature of the biofilm–antibiotic interactions taking place in the environment of drinking water supply lines, e.g., in poultry productions.

Concerning biofilm formation, co-culture biofilms were chosen because interspecies interactions may influence the outcome during antibiotic or disinfectant exposure experiments when compared with monoculture approaches [38,39,40]. The well-known biofilm formers pseudomonads (*P. aeruginosa* or *P. fluorescens*) were used due to their frequent detection in pipes of broiler [17] or pig premises [18], as well as due to their ability to aggregate rapidly and form biofilms [41]. Additionally, a reference strain of *E. coli* was included in the experiments as a representative of microbes, which are not usually part of water flora but may reach pipe networks via contamination with manure and pose a health risk for animals consuming water [18]. Based on the constant number of culturable bacteria after 7 days (Figure 1), the present biofilm growth method fulfilled quality requirements in terms of repeatability as well as uniformity for both co-culture biofilms, and therefore, seemed to be adequate for studying biofilm–antibiotic confrontation at an experimental level. Please note that microbiological counts for pseudomonads obtained through this approach were in an upper range for biofilm with *P. fluorescens* and slightly higher for biofilm with *P. aeruginosa* compared with those reported by Maes et al. [17] (counts of pseudomonads 0.6–6.1 log_10_ cfu cm^−2^) in drinking water systems of broiler houses in Belgium. The same applies to the data collected by Mateus-Vargas et al. [16] (counts of pseudomonads 2.3–3.3 log_10_ cfu cm^−2^) in models of drinking systems used in poultry productions. This is probably due to the higher concentrations of bacterial inoculum used for the experiments compared with the counts found in the water by the authors mentioned above [16,17]. Additional factors mentioned in the literature that may influence the bacterial counts in biofilms include biofilm age, flow rate, nutrient availability, as we all as substrate and biofilm’s microbial composition [6]. The bacterial density differed between biofilm formers and influenced the *E. coli* counts on PVC surfaces under nutrient-poor conditions. A more efficient biofilm-building capacity of *P. aeruginosa* is a plausible explanation for these observations. Under other growth conditions, counts of *P. aeruginosa* were higher than those of *P. fluorescens* [42,43]. As shown by Heydorn et al. [44], *P. aeruginosa* colonizes the available substratum more rapidly compared with *P. fluorescens* and other pseudomonad strains. Additionally, obvious differences in the counts of *E. coli* between both biofilm types in this study may be explained by competitive interactions. These may have led to growth hindrances in the forming biofilm, as previously reported with co-cultures of *P. aeruginosa* and *E. coli* [45].

Regarding antibiotic exposure of 7-day-old biofilms, experiments were performed using three antibiotics recommended for therapeutic application in drinking water: the combination of sulfadiazine/trimethoprim (SDZ/TMP), and tylosin A (TYL A). Effects of biofilm–antibiotic interactions were periodically monitored in the supernatant over 5 (SDZ/TMP for biofilm with *P. aeruginosa*) or 7 days (for all other experiments). We observed different dynamics between compounds when exposed to similar biofilm communities and conditions, in terms of substance concentration and the presence of transformation products. Although our observations were not completely foreseeable, we expected to observe contrasts due to obvious differences in physicochemical properties of the compounds used. TYL A belongs to the group of macrolides and is characterized by a high molecular weight (916.1 g mol^−1^). In contrast, SDZ and TMP both have appreciably lower molecular weights than tylosin (250.3 and 290.3 g mol^−1^, respectively). As shown in Figure 2, both SDZ and TMP seemed to be rapidly sequestered by biofilm structures in both biofilms although without suffering detectable changes at a molecular level. The latter is based on the observations made during the exposure period as well as the confirmation of residual concentrations in PBS-Tween samples at the end of the experiments. In the biofilm with *P. aeruginosa*, the diffusion/sorption of SDZ and TMP was stronger than in biofilm with *P. fluorescens*. Additionally, the concentration dynamics of TYL A in supernatants of pipes with *P. aeruginosa* was similar to the results of SDZ/TMP. In this case, TYL A seemed to interact differently with biofilm, since reductions in the supernatant were relatively lower and no residual concentrations were found at the end of the experiments. The latter may indicate, that TYL A was consistently washed out from surfaces during the sampling process and thus, entrapment by biofilm structures (if any) was not as strong as for SDZ or TMP. In contrast to other observations, the measured concentration of TYL A in BA with *P. fluorescens* and AC did not differ at day 0, decreased constantly over the complete period of exposure to biofilms, but was not detectable on surfaces containing biofilm (Figure 3). As shown by Lu et al. [46], molecular size affects the adsorption kinetics of tylosin (916.1 g mol^−1^) to different inert resins, compared with ciprofloxacin (331.5 g mol^−1^). In contrast, the comparable molecular size of tobramycin (467.5 g mol^−1^) and ciprofloxacin led to similar dynamics during the penetration of mucoid *P. aeruginosa* biofilms [47]. Therefore, for most of our observations, discrepancies in the concentration dynamics between SDZ/TMP and TYL A may be mainly explained by the obvious molecular weight differences. This may have influenced the diffusion/sorption in/by the biofilm matrix depending on the biofilm species and its specific matrix and architecture. Furthermore, based on their observations for tobramycin’s diffusion pattern in seaweed alginate beads, Cao et al. [48] implied that antibiotic concentration in biofilm may follow a power-law as a function of concentration in the surrounding liquid phase. Due to the different methodological approaches, it is unfortunately not possible to assess whether this effect is responsible for the rapid decrease at the beginning and the subsequent stabilization of the concentration levels in supernatant during the experiments.

Additional disparities in antibiotic dynamics during biofilm exposure may be related to differences in ionic charges of SDZ (partially anionic at pH 7) or TMP (most neutral, partially cationic) compared with TYL A (pKa of 7.7, most cationic) [49,50,51], since ionic interactions between biofilm matrix components and antibiotic molecules define the ability of antibiotics to penetrate the biomass [48,52]. Thus, the mostly cationic form of TYL A at neutral pH may have additionally affected its diffusion in biofilm. Further prediction models generating n-octanol-water partition coefficient K_ow_-values for hydrophobic interactions are unsuitable to explain our observations. By applying this model, TYL A, the substance with the highest partition coefficient of the tested analytes (SDZ logK_ow_ −0.09, TMP logK_ow_ 0.91, TYL A logK_ow_ 1.63) [53,54,55], would be expected to diffuse/to be absorbed into/by biofilm; however, this was just the case in our experiments in biofilms with *P. aeruginosa*. Similarly, sorption comparisons of erythromycin (logK_ow_ 0.98), sulfamethoxazole (logK_ow_ < −0.59) and ciprofloxacin (logK_ow_ −0.4) in biofilm reactors showed that, while sulfamethoxazole possessed the lowest, ciprofloxacin had the highest biofilm partition coefficient despite a lower K_ow_-value compared with erythromycin [56]. Therefore, in agreement with Wunder et al. [56], we suggest that the molecular size and antibiotic-specific characteristics (e.g., charge) may have played a major role in the antibiotic diffusion/sorption dynamics with biofilms of our current approach.

In this matter, it is important to consider differences in the biofilm formation capacities of both pseudomonads. Fazli et al. [57] reported clear differences in the EPS composition of biofilms produced by both pseudomonads. While the biofilm of *P. fluorescens* mostly contains larger surface proteins, EPS of *P. aeruginosa* was predominantly composed of exopolysaccharides (e.g., alginate, Psl, and Pel). Based on their observations with cationic antibiotics colistin and tobramycin and also anionic ciprofloxacin, Billings et al. [58] suggested that the polysaccharide Psl, which consists of repeating subunits of pentasaccharides, can entrap antibiotics in biofilm matrix via electrostatic interactions. Thus, such interactions may explain the different observations on TYL A dynamics between biofilms of both pseudomonads at the beginning of the trials (after 1 h), as well as the higher concentration difference of SDZ/TMP in biofilm with *P. aeruginosa* to controls compared with differences detected in experiments with *P. fluorescens*. Furthermore, regarding the pattern of decrease observed over the complete period of exposure for TYL A in biofilm with *P. fluorescens*, instead of sorption and sequestration, our results indicate that the decrease was caused by the transformation of the original substance by EPS and/or resident cells. Transformation strategies are part of the tolerance of biofilms against antimicrobial compounds due to the possibility of inactivation of antibiotic activity [6]. Known microbial transformation mechanisms include modifications by acylation, phosphorylation, glycosylation, nucleotidylation, ribosylation as well as thiol functional group transfer [59,60,61]. In our study, it was not possible to further characterize TP’s configuration due to its low stability in isolated solutions and, as such, we can only speculate about mechanisms that underlie our observations. For instance, hydrolysis of the lactone ring by macrolide esterases, which results in the cleavage of macrocycle has been described for erythromycin [62,63]. In our case, we assume that TYL A was not hydrolyzed by an esterase, since TP turned quickly back to TYL A in the isolated solutions. Further mechanisms such as phosphorylation or glycosylation taking place on the free hydroxyl-site (site 2′) are described for macrolides [59]. Glycosylation is a transformation strategy used by macrolide producers and not yet reported in intrinsic or opportunistic pathogens with exception of some strains of *Nocardia* species [64,65,66]. In contrast, macrolide 2′-phosphotransferases (mphs) have been identified in clinical strains of *Staphylococcus aureus*, *E. coli,* and *P. aeruginosa* phosphorylate 14- and some 16-membered ring macrolides [67,68,69]. Indeed, the transformation of tylosin in tylosin-2′-phosphate was firstly verified by using an isolated enzyme of *Streptomyces coelilor* [70,71]. Although we cannot completely discard the actual presence of such a transforming enzyme, to the best of our knowledge, the production of phosphotransferases has not been described for the strains used in our experiments. It is noteworthy that the transformation rate of TP represented only 0.8% while the decrease in the concentration of TYL A reached up to 3.4%. The observed incongruence may be due to different extinction coefficients, reduced absorption at 280 nm of the detected TP, or the presence of other transformation products that may not absorb in the measured UV range (250–350 nm) [72]. HPLC has to be precisely adjusted to adequately separate the peaks belonging to the different tylosin derivates. The baseline separation of the peaks by analytical reversed-phase HPLC was a big challenge (Figure 4). Strategies such as the injection of higher sample volumes or a longer biotransformation time to obtain higher amounts of TP in the solution were unsuccessful. The latter is because TYL A and related substances eluting around TP likewise reached higher concentrations in the solutions and consequently, no baseline separation of the peaks was possible, even with optimized HPLC parameters. Concentration and purification in the solid phase extraction (SPE) were comparably fruitless due to the similar elution behavior of tylosin derivates. It is moreover important to point out that measured decreases in antibiotic concentrations in supernatants were at least in some cases statistically significant. Thus, interpretation of these results should be made with great caution. In our case, statistical models show a relative consistency of the observations between trials but do not relate to the degree of dissimilarity between concentrations in pipes with or without biofilm.

Regarding biofilm development, bacterial counts of biofilms showed that the density of culturable cells on PVC surfaces was differently affected by antibiotic exposure (Figure 1). The first noticeable finding of our experiments was that bacterial counts of biofilms exposed to SDZ/TMP remained comparable to those of controls. Since surviving *E. coli isolates* were phenotypically susceptible to SDZ/TMP as well as to a range of other antibiotic substances after exposure (Table 1), it is likely that the observed tolerance of sessile *E. coli*-cells is specifically related to characteristics proper of biofilm state. Numerous studies have reported that biofilm tolerance mechanisms are mostly due to hindrance of antibiotic penetration by extracellular matrix and/or to the metabolic activity of cell subpopulations [6]. Interestingly, chemical analyses of our study suggest that at least some biofilm-forming cells were in proximity to undistorted antibiotic molecules since important residual concentrations were at the end of the experiment still detectable in biofilm samples. Concerning in vitro studies of biofilms of *P. aeruginosa*, increased tolerance has been attributed to slower growth rates and cellular activity of sessile microorganisms related to oxygen limitation [47,73,74], and starvation [75], both favored by the properties of the biofilm micro-environment. Moreover, not only competitive but also protective interactions are observed in multispecies biofilms during antibiotic confrontation performed in vitro [40]. Interestingly, Wang et al. [20] reported that sulfadiazine exposure of biofilms in urban drinking water distribution systems resulted in higher production of EPS, which permitted even the increase of total bacterial counts in biofilms. Hence, and together with the experimental conditions [76], the mentioned characteristics of the co-culture biofilms of the present study may have contributed to the effective survival of sensitive bacterial cells against the applied concentrations of SDZ/TMP.

The second interesting observation of the study concerning sessile bacteria was that the 7-day application of TYL A resulted in a general decrease of bacterial counts in biofilms compared with controls. Tylosin is a bacteriostatic antibiotic used in veterinary medicine to control respiratory diseases caused by Gram-positive pathogens and Mycoplasma species [77] but has low bactericidal activity against Gram-negative bacteria [78]. The latter is due to the effective protection conferred by the outer membrane of Gram-negative bacteria against macrolide’s transmembrane diffusion [79]. However, macrolides have also been reported to inhibit the synthesis of exopolysaccharides and affect consequently the structural configuration of biofilms formed by Gram-positive bacteria like *Streptococcus suis* [80] as well as Gram-negative like *P. aeruginosa* [81] or *Salmonella enterica* serovar Typhimurium (*S*. Typhimurium) [82]. Moreover, in vitro studies have also shown synergistic antibacterial effects between tylosin and active compounds of some plant extracts against the Gram-negative *S*. Typhimurium [82,83]. Due to the minimal growth conditions in our experiments, it is rather unlikely that reduced bacterial loads of exposed biofilms are due to the combined antibacterial activity of tylosin and other active compounds. Therefore, consistent with the observations mentioned above, the applied concentrations of tylosin may have interfered with bacterial EPS production and consequently led to a decreased number of culturable bacteria attached to the PVC surface compared with unexposed bacterial controls. Based on the chemical analyses, it is plausible that biofilm inhabitants may activate transformation mechanisms (e.g., enzymes) as a mode of action to reduce the impact of TYL A on biofilm [81,84].

Moreover, susceptible *E. coli* in both biofilms were able to survive being exposed to SDZ/TMP. Based on the chemical observations, the surrounding biofilm and/or the sessile metabolic state presumably conferred protection against noxious antibiotic concentrations. The latter would be probable at least for the inhabitants of the periphery of the sensitive bacteria populating the biofilm [35,52]. Furthermore, the results of susceptibility testing suggest that the selective pressure imposed by a single application of these authorized veterinary drugs may not be sufficient for the development of resistant bacteria in water biofilms. This was expected to be triggered by mutation episodes in this case [84]. Although our results indicate that a single treatment may have little or no significance for the development of resistance among sensitive bacteria, under real-life conditions, the surviving cells would further be confronted repeatedly with the same or other chemicals over long periods. The latter are good prerequisites for the emergence and stabilization of resistance through mutation processes [85]. However, since long exposures to trace levels of antibiotics did not change the antimicrobial susceptibility of bacteria from drinking water distribution systems [18], the role of the biofilm–antibiotic interactions during the medication with drinking water in poultry farms remains unclear.

## 4. Materials and Methods

### 4.1. Chemicals

Compounds included authorized veterinary drugs containing the combination sulfadiazine/trimethoprim (SDZ/TMP; Trimetotat, aniMedica GmbH, Bösensell, Germany), and tylosin A (TYL A; VETRANAL™, Sigma-Aldrich, Germany). These antimicrobials seemed to be adequate due to their frequent use in food-producing animals in Europe [77], as well as the obvious structural differences at the molecular level between all substances. Standards for analysis of sulfadiazine, trimethoprim, and tylosin A (VETRANAL™ grade, SDZ/TMP purity ≥ 99.0%, TYL A: tylosin tartrate, charge-dependent: 85–92%) were obtained from Sigma-Aldrich (Merck KGaA, Darmstadt, Germany). The pure standards for the qualification of Tylosin A, B, C, and D were from Laboratorium Farmaceutische Analyse, Leuven (Belgium). For nutrient-poor media preparation, glycerol, L-glutamine, and K_2_HPO_4_ were obtained from Carl Roth GmbH + Co. KG (Karlsruhe, Germany); MgSO_4_ was purchased from Thermo Fisher Scientific, (Kandel, Germany). For phosphate-buffered solution (PBS) with tenside, NaCl, KH_2_PO_4_, Na_2_HPO_4_ ∙ 2 H_2_O were obtained from Merck Millipore (Merck KGaA, Darmstadt, Germany); KCl and Tween^®^ 20 from Carl Roth GmbH + Co. KG (Karlsruhe, Germany).

HPLC-grade methanol and acetonitrile were obtained from J. T. Baker (Avantor Performance Materials, Gliwice, Poland). Formic acid and ammonium acetate were acquired from Bernd Kraft (Duisburg, Germany) and Merck (Darmstadt, Germany), respectively.

### 4.2. Strains and Culture Conditions

For the culture of biofilms, PVC pipe pieces (PVC-U; DIN 8061: diameter 40.0 mm, length 100.0 mm) were thoroughly cleaned, dried, and submerged in ethanol 70% for 5 min for disinfection before conducting experiments. To establish a bacterial co-culture, two to three colonies obtained from overnight cultures of *Pseudomonas aeruginosa* (ATCC 27853), *P. fluorescens* (ATCC 13525), and *E. coli* (ATCC 23631) were separately suspended in a nutrient-poor medium (1.9% glycerol, 0.5% L-glutamine, 0.15% K_2_HPO_4_, and 0.01% MgSO_4_; pH 7.0). The turbidity of the suspensions was adjusted to 0.125 absorbance at 600 nm (BioDrop, Biochrom Ltd., Cambridge, UK). Equal volumes of the suspensions were mixed to obtain a combination of a pseudomonad species (either *P. aeurginosa* or *P. fluorescens*) and *E. coli* for further experiments (stock suspension). For inoculation, each pipe was closed at one end with a PVC-threaded cap, placed upright, and then filled with 50 mL of final bacterial suspension (1.5 to 3.0 × 10^8^ cfu mL^−1^). For each experiment, the concentration of bacterial stock suspensions was confirmed by plate counting. Thereafter, pipes were stored at 22 °C ± 1 °C for 7 days to allow biofilm development on the inner surface of the pipes. To improve oxygenation of media, pipes were carefully shaken for 2 min on a daily basis during the development period.

### 4.3. Experimental Design

The veterinary drugs were diluted in nutrient-poor medium to the concentrations recommended for therapeutic application in drinking water (SDZ/TMP: 150/30 mg L^−1^; TYL A: 500 mg L^−1^). Prior to antibiotic application, old media in pipes were poured off, and every pipe was gently washed three times each with 50 mL water (0.9% NaCl) to remove non-adherent cells. After washing, the pipes were filled with the antimicrobial solution and stored at 22 °C ± 1 °C for 5 (SDZ/TMP in biofilm with *P. aeruginosa*) or 7 (SDZ/TMP in biofilm with *P. fluorescens* and TYL A in both biofilms) days, which is close to the recommended treatment period (http://www.vetidata.de, accessed on 13 August 2021). This storage temperature was chosen for the pipes accordingly to recommendations for the inside air temperature of barns used to house broiler chickens older than 4 weeks [86]. Additionally, pipes containing biofilm were filled with medium without antibiotics as a biofilm development control. Furthermore, the clean pipes were filled with antimicrobial solutions and stored under similar conditions to monitor compound behavior without contacting biofilm. The experiments were conducted in three sets to determine (1) the effect of antibiotic administration on biofilm community composition and to monitor (2) the effect of biofilm on the antibiotic bioavailability as well as on the chemical composition of the antimicrobial compounds during the confrontation. Microbiological examinations were performed before and after antibiotic exposure. For chemical examinations, sampling started 1 h after antibiotic application and continued daily during the confrontation period. At the end of the experiments, sample suspensions obtained from pipe surfaces were examined to determine antimicrobial residual concentrations. For all chemical analyses, samples were sterile-filtered (0.2 µm, polyester membrane material) after collection and stored at 4 °C until all investigations were performed.

### 4.4. Chemical Analysis

The concentrations of SDZ/TMP and TYL A in the supernatants obtained daily from pipes with and without biofilm were determined by HPLC-UV. Additionally, PBS-Tween samples obtained at the end of the experiments (microbiological analyses) were tested for the presence of residual concentrations of antibiotics on the inner PVC surfaces by HPLC-UV. For the measurement of the combination SDZ and TMP, a Hypersil^TM^ GOLD column (150 × 4.6 mm, 5 μm; Thermo Scientific, Dreieich, Germany) was employed. Analytes were eluted with a binary gradient of 1 mM ammonium acetate in 0.5% formic acid (A) and methanol (B) as follows: start with 15% B; 0−6 min, ramp to 45% B; 6−7.5 min, ramp to 80% B; 7.5−8.5 min, hold at 80% B; 8.5−9.5 min, ramp to 15% B; 9.5−14 min, hold at 15% B. All parts of the HPLC system were from Shimadzu, Japan. The SIL-10ADvp autosampler was used to inject 10 µL of the sample into the HPLC system and an LC-10ADvp low-pressure pump maintained a constant flow rate of 1 mL min^−1^. For separation, the temperature was kept at 30 °C in a CTO-10ACvp oven. Detection was performed at 270 nm using an SPD-10Avp UV detector combined with Class-VP software (version 6.13, Merck-Hitachi, Darmstadt, Germany). For quantification, a calibration curve for SDZ and TMP was obtained by linear regression corresponding to the correlation between the peak area and the analyte concentration ranging from 100 to 200 mg L^−1^ and 10 to 50 mg L^−1^ for SDZ and TMP, respectively. The limit of detection (LOD) and limit of quantitation (LOQ) were calculated using the calibration method from the German standard method [23].

For the measurement of TYL A, HPLC equipment mentioned above was used in conjugation with a Hypersil^TM^ GOLD PFP column (150 × 4.6 mm, 5 μm; Thermo Scientific). Prior to measurement, the samples treated with TYL A were diluted 1 to 5 with high purity water and sterile filtered (0.2 µm, polyester as membrane material). 20 µL of the diluted samples were injected, and subsequently eluted with a tertiary gradient with mixed eluents: the basic component buffer for the mixed eluents was 1 mM ammonium acetate in 0.5% formic acid in high-purity water. Eluent (A) consisted of 52% buffer solution and 48% methanol, (B) 75% buffer solution and 25% acetonitrile, and (C) pure acetonitrile. The gradient started with 100% A for 0.1 min, 0.1–3.0 min, ramped to 53% A and 47% B; 3.0–11.7 min, ramped to 100% B; 11.7–15.0 min, ramp 87% B and 13% C; 15.0–16.6 min, ramp to 73% B and 23% C; 16.6–18.2 min, ramp to 100% A; 18.2–23.0 min hold at 100% A. The pump guaranteed a constant flow rate for 19.7 min at 1 mL min^−1^, then 3.1 min at 1.5 mL min^−1^, followed by a subsequent decrease to 1 mL min^−1^ for 23.0 min. For TYL A, the detection wavelength was 280 nm and the oven temperature was kept at 25 °C. For the quantification of TYL A, a calibration curve was generated within an analyte concentration of 50 to 150 mg L^−1^. Furthermore, the isolation of transformation products was achieved by using different variations of the HPLC method described above.

### 4.5. Microbiological Analysis

Biofilm growth on inner PVC surfaces was controlled before and after the antibiotic exposure period through the plating method. Briefly, the old medium was poured off and each pipe was gently washed three times with 50 mL water (0.9% NaCl) to remove non-adherent cells. Thereafter, the complete inner surfaces of the PVC pipes were thoroughly wiped with 2 sterile swabs, consecutively. Thereafter, swab samples were collected in 50 mL of 0.01% PBS-Tween20 contained in 50 mL centrifuge tubes and thoroughly homogenized by vortexing for 1 min. Bacterial load in biofilms was assessed by plating serial dilutions in triplicate on the surface of McConkey agar (CM01105; Oxoid GmbH, Wesel, Germany) for *E. coli* (24 h; 36 °C) and Pseudomonas CFC agar-plates (Oxoid GmbH) for pseudomonads (24 h; 30 °C). Following incubation, all colonies showing typical characteristics were counted to determine the number of colony-forming units (cfu) per mL. Biofilm results were further normalized to cfu per surface area (cfu cm^−2^). After quantification, two isolates of *E. coli* from each pipe were randomly selected for antimicrobial susceptibility testing, resulting in 6 isolates for each type of antimicrobial application. From the control biofilms, 12 isolates were chosen for antimicrobial susceptibility testing.

### 4.6. Antimicrobial Susceptibility Testing

The susceptibility of the isolates was determined using a broth microdilution technique (Sensititre, Trek Diagnostic Systems, West Sussex, United Kingdom) for 12 antimicrobial substances with plates especially costumed for this study (TREK Diagnostic) (Table 1), in accordance with CLSI procedures [87]. Antimicrobial substances are representative of the major classes of antimicrobial drugs and have been recommended by the European Food Safety Agency for monitoring and reporting antimicrobial resistance in *E. coli* from food animals [88]. Following 24 h of incubation at 36 °C, the plates were read using VizionTM digital viewing system (Trek Diagnostic Systems). Separately, susceptibility against TYL A was determined using the broth microdilution method in 96-well plates. Briefly, stock solutions of TYL A (50 mg L^−1^, 2.5% *v*/*v* methanol, 0.1 M phosphate buffer, pH 7) were serially two-fold diluted in Mueller-Hinton broth (Carl Roth GmbH + Co. KG, Karlsruhe, Germany) and distributed into 96-well plates (180 µL per well) to obtain a final concentration per well ranging from 2 to 2048 mg L^−1^. The *E. coli* isolates were subcultured on 7% Columbia sheep blood agar plates (Thermo Fisher Scientific, Waltham, MA, USA) for 24 h at 37 °C. Two to three colonies of each isolate were separately suspended in 0.9% NaCl and turbidity was adjusted to 0.08 absorbance at 600 nm (Biochrom WPA CO8000, Biochrom Ltd., Cambrindge, UK). Each *E. coli* suspension was further diluted in Mueller-Hinton broth at a ratio of 1:100 and then 20 µL were inoculated in each well to reach a final concentration of ~10^4^ cfu mL^−1^ per well, which was confirmed for each set by plate counting. After 24 h at 36 °C, the turbidity was evaluated at 570 nm using an MRX-reader (Dynatech, Denkendorf, Germany). For both microdilution approaches, the minimum inhibitory concentration (MIC) was read as the lowest concentration of the antimicrobial capable of inhibition of bacterial growth and were interpreted according to the epidemiological cut-offs (ECOFF) published by the European Committee on Antimicrobial Susceptibility (EUCAST) [22]. Additionally, the MIC for 50% (MIC_50_) and 90% (MIC_90_) of all isolates tested was analyzed to compare the MIC distributions of the exposure to those of control biofilms. The antimicrobial compounds used, concentration range tested, and breakpoints are presented in Table 1. *E. coli* ATCC 25922 was used as a reference for the quality control of susceptibility tests. Pseudomonads were excluded from antimicrobial susceptibility testing because of their high intrinsic resistance [89] and the lack of published ECOFFs for most of the antibiotics tested [22].

### 4.7. Statistical Data Analysis

For conduction of analysis, resulting bacterial counts (cfu cm^−2^) were converted into log_10_-values. Furthermore, the percentage values for antibiotic concentration in supernatants were calculated by comparing the concentration (mg L^−1^) of substances in media with biofilm confrontation to the average concentration of controls at day 0 (%). Results are presented as mean ± standard deviation (SD). Statistical analyses were performed with GraphPad Prism (version 9.1.0, GraphPad Software, Inc., San Diego, CA, USA). Comparisons of bacterial counts and antibiotic concentrations between exposed biofilms and controls were performed with the multiple unpaired *t*-tests and the Mann–Whitney U test. The level of significance was set at *p* < 0.05.

## 5. Conclusions

In conclusion, a reliable and rugged model approach enabled complementary evaluations from a chemical and microbiological perspective. This study allowed testing initial hypotheses on possible interactions between antibiotics and bacterial biofilms under conditions present in water distribution systems, e.g., those of poultry farms. Although only relatively small concentration decreases were observed for all antibiotics under investigation due to contact with biofilms, our results indicate that antimicrobial compounds may interact with biofilms under premise conditions and affect bioavailability during antibiotic administration. Since the TP of TYL A was stable in the sample solution over weeks (data not shown), a constant presence of this TP in water pipes at least during the medication period may be assumed. Comparing the interactions of biofilms between two different pseudomonads, we show the important role of bacterial composition in the assessment of processes on various levels taking place during interactions. Due to the variability in biofilm’s bacterial composition in water systems observed between broiler productions [17], it is still not possible to make any further general assumptions about the final effects of biofilm–antibiotic interactions on animals consuming water. In real life, such interactions may simultaneously trigger microbial processes that could mutually complement each other. Additionally, the biotransformation of macrolides (azithromycin, erythromycin, and clarithromycin) caused by activated sludge biomass result in transformation products with reduced antibiotic activity and lower algal toxicity compared with the original compounds [90]. Thus, the transformation strategies of biofilms populating drinking water systems should be considered during antibiotic administration via drinking water. Additionally, the results from susceptibility testing *E. coli* isolates suggest that a single antibiotic exposure had no immediate measurable effect on the development of resistant bacteria in 7-day-old biofilms. Survival of *E. coli* in biofilms shows that potentially pathogenic fecal bacteria may not be reduced in the biofilms from drinking systems after antibiotic application. Due to the potential role of established biofilms in the environmental persistence of important pathogens such as *Campylobacter jejuni* [91] or *Actinobacillus pleuropneumoniae* [92], our observations underline the importance of biosecurity measures in animal farms, including adequate cleaning and disinfection procedures, to break infection chains within and between flocks. Finally, since the efficient use of antimicrobial compounds in livestock is a fundamental measure to confront the continuous reduction of the therapeutic efficacy of antimicrobials [2], future studies should further assess if water medication is an adequate treatment option in animal husbandry. The suitability of the presented approach for evaluating the administration of veterinary medicine via drinking water may benefit from further optimization. Future experimental settings should be sure to include different hydrodynamic conditions that may better reflect conditions present in poultry drinking water systems. Furthermore, researchers should attempt to characterize both antimicrobial resistance and biofilm response using advanced methods, such as proteomics, metabolomics, and fluorescence microscopy. Investigating the possible effects of transformation products on treatment success, toxicology, as well as antimicrobial resistance development in livestock represents a major challenge for future research.

## Figures and Tables

**Table 1:** Antimicrobials tested, concentration range, reference concentrations and distribution of MIC values (mg L^−1^) among *E. coli* isolates (*n* = 20) obtained from biofilms after antibiotic exposure.

Antimicrobial ^1^	Concentration Range	Epidemiologic Cut-Off ^2^	MIC_50_	MIC_90_
AMP	0.25–32	≤8	4	4
CTX	0.06–8	≤0.25	≤0.06	≤0.06
CAZ	0.06–8	≤0.5	0.25	0.5
CHL	2–128	≤16	8	8
CIP	0.015–2	≤0.06	≤0.03	≤0.03
COL	0.12–16	≤2	1	2
GEN	0.25–16	≤2	1	2
NAL	1–128	≤16	4	8
SUL	2–256	-^3^	4	4
TET	0.25–32	≤8	2	2
TMP	0.25–32	≤2	≤0.25	≤0.25
TYL A	2–2048	-^3^	1024	1024
SDZ/TMP	0.59/0.03–76/4	≤19/1	≤0.59/0.03	≤0.59/0.03

^1^ Abbreviations describe antibiotic substances as follows: ampicillin (AMP); cefotaxim (CTX); ceftazidim (CAZ); chloramphenicol (CHL); ciprofloxacin (CIP); colistin (COL); gentamicin (GEN); nalidixic acid (NAL); sulfisoxazole (SUL); tetracycline (TET); trimethoprim (TMP); tylosin A (TYL A); sulfadiazine/trimethoprim (SDZ/TMP). ^2^ Epidemiologic cut-off published by the European Committee on Antimicrobial Susceptibility (EUCAST) [22]. ^3^ No epidemiologic cut-off value was reported by EUCAST [22].

## Data Availability

The data presented in this study are available on request from the corresponding author. The data are not publicly available due to the large data set.
